# Paeoniflorin alleviates hypoxia/reoxygenation injury in HK-2 cells by inhibiting apoptosis and repressing oxidative damage via Keap1/Nrf2/HO-1 pathway

**DOI:** 10.1186/s12882-023-03366-0

**Published:** 2023-10-26

**Authors:** Di Xing, Yihua Ma, Miaomiao Lu, Wenlin Liu, Hongli Zhou

**Affiliations:** 1 Department of Nephrology, The First Affiliated Hospital of Jinzhou Medical University, Liaoning, China; 2 Department of Traditional Chinese Medicine, The First Affiliated Hospital of Jinzhou Medical University, Liaoning, China

**Keywords:** Paeoniflorin, Hypoxia/Reoxygenation Injury, Oxidative stress, Nrf2/HO-1 pathway

## Abstract

Acute kidney injury (AKI) is a serious disorder associated with significant morbidity and mortality. AKI and ischemia/reperfusion (hypoxia/reoxygenation, H/R) injury can be induced due to several reasons. Paeoniflorin (PF) is a traditional herbal medicine derived from *Paeonia lactiflora* Pall. It exerts diverse therapeutic effects, including anti-inflammatory, antioxidative, antiapoptotic, and immunomodulatory properties; thus, it is considered valuable for treating several diseases. However, the effects of PF on H/R injury-induced AKI remain unknown. In this study, we established an in vitro H/R model using COCL_2_ and investigated the functions and underlying mechanisms of PF on H/R injury in HK-2 cells. The cell vitality was evaluated using the cell count kit-8 assay. The DCFH-DA fluorescence probe was used to measure the levels of reactive oxygen species (ROS). Oxidative damage was detected using superoxide dismutase (SOD) and malondialdehyde (MDA) assay kits. Apoptotic relative protein and Keap1/Nrf2/HO-1 signaling were evaluated by Western blotting. Our results indicated that PF increased cell viability and SOD activity and decreased the ROS and MDA levels in HK-2 cells with H/R injury. PF inhibits apoptosis by increasing Bcl-2 and decreasing Bax. Furthermore, PF significantly upregulated the expression of HO-1 and Nrf2, but downregulated the expression of HIF-1α and Keap1. PF considerably increased Nrf2 nuclear translocation and unregulated the HO-1 expression. The Nrf2 inhibitor (ML385) could reverse the abovementioned protective effects of PF, suggesting that Nrf2 can be a critical target of PF. To conclude, we found that PF attenuates H/R injury-induced AKI by decreasing the oxidative damage via the Nrf2/HO-1 pathway and inhibiting apoptosis.

**Supplementary Information:**

The online version contains supplementary material available at 10.1186/s12882-023-03366-0.

## Introduction

Acute kidney injury (AKI) is responsible for a considerably huge financial burden on society, including significant morbidity and mortality rates. Several conditions can cause AKI, and ischemia/reperfusion injury (IRI) is one of them. IRI is characterized by the interruption of blood to an organ for a certain time, followed by blood flow and oxygen recovery [1], which can occur after kidney transplantation, sepsis, and cardiac surgery [2]. The kidney is a hyperperfusion organ, which is susceptible to IRI after the brain and heart [3]. IRI can lead to several pathological phenomena, including oxidative stress, inflammatory reaction, and apoptosis [2]. In the ischemic stage, the organism is under a hypoxic environment, that is devoid of adequate erythrocytes delivering oxygen. This situation suppresses mitochondrial oxidative phosphorylation and then impairs the activity of cellular energy-dependent processes due to a lack of ATP [1], [3]. When the blood is restored in the reperfusion phase, reactive oxygen species (ROS) are produced, which lead to protein oxidation, lipid peroxidation, and DNA damage [3]–[4].

Paeoniflorin (PF) is a traditional herbal medicine isolated from *Paeonia lactiflora* Pal [5] with various biological implications, such as antioxidative [6], anti-inflammatory, antiapoptotic [7], analgesic [8], and immunomodulatory properties [9]. Previous studies have indicated that PF exerts protective effects on the heart, brain, kidney, and liver from myocardial infarction [10], diabetic nephropathy [11], and stroke [12]. Several previous studies have indicated that PF can protect the heart, brain, and liver from IRI [10], [12]–[13]. Recently, Jin Wen et al. reported that PF mitigated intestinal IR-impaired autophagy flux by activating the LKB1/AMPK signaling pathway [14]. Furthermore, Chinese researchers have reported that PF could ameliorate acute necrotizing pancreatitis-induced AKI by inhibiting inflammation and renal cell apoptosis [15]. Nevertheless, the role of PF on I/R-induced AKI and the potential mechanism remain unknown.

Nuclear factor-E2-related factor 2 (Nrf2) is considered a critical transcription factor in the antioxidative system, which combines with Keap1 and enters proteasomal degradation via Keap1-mediated ubiquitination in normal conditions [16]. When the organism is stimulated by endogenous or exogenous factors that result in oxidative stress, the negative regulation of Keap1 on Nrf2 is attenuated. Nrf2 cannot bind to Keap1 and becomes free and enters the nucleus and combines with antioxidant reaction elements (ARE) to induce the formation of antioxidative enzymes, such as NQO-1, HO-1, and GPX4^17^. Jing Yu et al. reported that PF can alleviate oxidative damage by initiating the Nrf2/HO-1 pathway in gamma-radiation-treated human EA.hy926 endothelial cells [6]. A new study indicated that Nrf2 plays a major role in protecting the kidneys from oxidative damage [17]. The study reported that the activation of Nrf2 with CDDO-imidazolide protects against AKI by improving survival and renal functions in IRI mice [18].

In this study, we constructed a hypoxia/reoxygenation (H/R) model in HK-2 cells to mimic the development of IRI and investigated the effects of PF and its underlying mechanism on AKI.

## Materials and methods

### Reagents

Cobalt chloride (COCL_2_, purity ≥ 99%) was purchased from Jingshiji (Changsha, China). PF (purity ≥ 98%) and 2’7’-dichlorofluoresceine diacetate (DCFH-DA) were purchased from Solarbio Life Sciences (Beijing, China). Cell Counting Kit-8 (CCK-8) was purchased from Glpbio (USA). Superoxide dismutase (SOD) assay kit and malondialdehyde (MDA) assay kit were bought from Nanjing Jiancheng Bioengineering Institute (Nanjing, China). Mouse-anti-β-actin was purchased from Servicebio (Jiangsu, China). Rabbit anti-hypoxia-inducible factor-1 α (HIF-1α) was purchased from Abcam (Cambridge, England); rabbit anti-Nrf2 and HO-1 from Proteintech Group, Inc. (Wuhan, China); rabbit anti-Keap1 from Beyotime Biotechnology, Inc. (Beijing, China); rabbit anti-Lamin B from Wanleibio Inc. (Shenyang, China); rabbit anti-Bax and Bcl-2 from Cell Signaling Technology Inc. (Danvers, Massachusetts, USA.)

### Cell culture and treatment

Human proximal tubular epithelial cells (HK-2) were cultured in DEME/F12 (Hyclone, USA) with 10% fetal bovine serum (FBS, Every Green, Zhejiang China) and 1% penicillin–streptomycin (Procell, Wuhan, China). HK-2 cells were placed in an incubator (SANYO, Japan) at a temperature of 37 °C under a 5% CO_2_ humidified atmosphere. PF treatment was administered by adding PF to DEME/F12 medium and achieving a final concentration of 200 µM. Nrf2 was inhibited by adding ML385 (APE, USA) into DEME/F12 to a final concentration of 5 µM.

### HK-2 cell H/R model and experiment design

COCL_2_, a chemical hypoxia inducer [19], was added to a serum-free culture medium for 24 h to mimic the ischemia phase, and reoxygenation was achieved by changing DEME/F12 medium supplemented with 10% FBS for 4 h in the incubator to mimic the reperfusion phase.

The HK-2 cells were divided into the following 4 groups: untreated HK-2 cells cultured in DEME/F12 containing 10% FBS (NC group), hypoxia/reoxygenation model (H/R group), HK-2 cells incubated with PF for 4 h before H/R induction (H/R + PF group), and ML385 added to DEME/F12 for 24 h, followed by treatment as the H/R + PF group (H/R + PF + ML385 group).

### Measurement of cell viability

Cell counting kit-8 (CCK-8) was used to evaluate the cell viability. HK-2 cells were added to a 96-well culture plates overnight at a density of 3,000 cells/well to adhere overnight, then were treated with COCL_2_ (0–1200 µM) for 24 h in the incubator. PF was added into the culture medium at different concentrations (50 µM, 100 µM, and 200 µM) for 4 h before H/R treatment. After changing the fresh culture medium, 10 µL of CCK-8 (Glpbio, USA) was added into each well and incubated for 2 h in the dark. The absorbance was determined and recorded at 450 nm wavelength using a microplate reader (Infinite M200 PRO, TECAN, Switzerland). Cell viability was calculated as follows:

Cell viability (%) = (OD treatment – OD blank) / (OD control – OD blank) ×100.

### Measuring intracellular reactive oxygen species

Intracellular reactive oxygen species (ROS) was measured using an oxidation-sensitive fluorescent probe (DCFH-DA). HK-2 cells were pretreated with ML385 (5 µM) for 24 h and then with PF (50–200 µM) for 4 h followed by H/R treatment. After H/R treatment, each well was added with serum-free culture medium containing 10 µM of DCFH-DA and incubated at 37 °C for 20 min. After washing thrice with serum-free medium, the cells were collected for flow cytometry analysis or directly observed using a fluorescence microscope (OLYMPUS IX51, Japan). Finally, the cells were photographed in three randomly selected fields (×200 magnification).

### Detection of MDA level and SOD activity

The MDA level and SOD activity were determined using the malondialdehyde assay kit (Nanjing Jiancheng, China) and the superoxide dismutase assay kit (Nanjing Jiancheng, China) respectively. The cells were collected in a centrifugal tube after the relative treatment of each group and then detected as per the manufacturer’s instruction. The absorbance was detected at a wavelength of 532 nm and 450 nm, respectively, using a microplate reader (Infinite M200 PRO, TECAN, Switzerland). The SOD activity was expressed as units/mg of protein and the MDA level was expressed as nanomoles/mg of protein.

### Western blotting

RIPA buffer (Beyotime, Beijing, China) added with protease inhibitor was used to extract total protein. A nuclear and cytoplasmic protein extraction kit (Wanleibio, Shenyang, China) was used to extract the nuclear and cytosolic proteins per the manufacturer’s instructions. The protein concentration of each group was evaluated using the bicinchoninic acid (BCA) protein assay kit (Glpbio, USA). The equal mass of protein (30 µg) taken from each group was separated using 10% sodium dodecyl sulfate (SDS)–polyacrylamide gel electrophoresis (PAGE) and then transferred to polyvinylidene difluoride membranes (Millpore, Ireland). The membranes were blocked using 5% skimmed milk at room temperature for 2 h, cut into corresponding parts according to the marker, and then incubated respectively with rabbit anti-HIF-1α (1:2000, Abcam), rabbit anti-Nrf2 (1:2000, Proteintech), rabbit anti-Keap1 (1:2000, Beyotime), rabbit anti-HO-1 (1:2000, Proteintech), rabbit anti-Bcl-2 (1:2000, CST), rabbit anti-Bax (1:2000, CST), mouse anti-β-actin (1:3000, Servicebio), and rabbit anti-Lamin B1 (1:1000, Wanleibio) primary antibody for overnight at 4 °C. After washing thrice with tris-buffer saline tween (TBST), the membranes were incubated with goat anti-mouse IgG H&L (1:3000, Wanleibio) or goat anti-rabbit IgG H&L (HRP) (1:5000, Wanleibio) at room temperature for 1 h. The membranes were washed thrice with TBST and processed for visualization using the Ultra High Sensitivity ECL Kit (Glpbio, USA) and detected using a Luminescent Image analyzer. The protein bands were analyzed using ImageJ software.

### Statistical analysis

The continuous variable is expressed as the mean ± SD. GraphPad Prism 8 was used to statistically analyze the dates with a one-way analysis of variance (ANOVA) following Tukey’s post-hoc test. All statistical tests were considered significant when the two-tailed *p*-value was < 0.05.

## Results

### PF increased the viability of HK-2 cells after H/R treatment

**Fig. 1:**
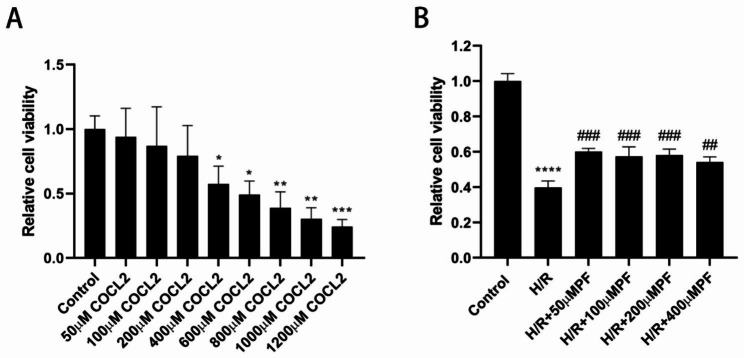
The influences of COCL_2_ and PF on cell viability of HK-2 cells. (**A**) The cell vitality of HK-2 cells after COCL_2_ administration. (**B**) The effects of PF on the cell viability of HK-2 cells after H/R injury. Cell vitality was detected by performing CCK-8 assays. Cell viability of the COCL_2_-treated groups and other experimental groups were expressed as a percentage of the viability in the control group. Data are presented as the mean ± SD (n = 3). *p < 0.05, **p < 0.01, ***p < 0.001, ****p < 0.0001, in comparison with the control group. ####*p* < 0.0001, in comparison with the H/R group. H/R, hypoxia/reoxygenation, PF, paeoniflorin, SD, standard deviation

To investigate a suitable concentration of COCL_2_-induced HK-2 cell hypoxic environment, we set a series concentration of COCL_2_ (0–1200 µM) and evaluated it after 24 h of COCL_2_ treatment. Administration with COCL_2_ (400–1200 µM) considerably decreased cell viability (*p* < 0.05), among which the cell viability in the 400-µM COCL_2_ group was 57.53 ± 11.26% (Fig. 1A). Therefore, 400-µM COCL_2_ was selected as the final concentration in the subsequent experiment. Moreover, PF increased the cell viability of HK-2 cells with H/R injury (Fig. 1B), but exhibited no significant distinction in the three different concentrations of the PF group.

### PF attenuated ROS production in H/R-induced HK-2 cells

**Fig. 2:**
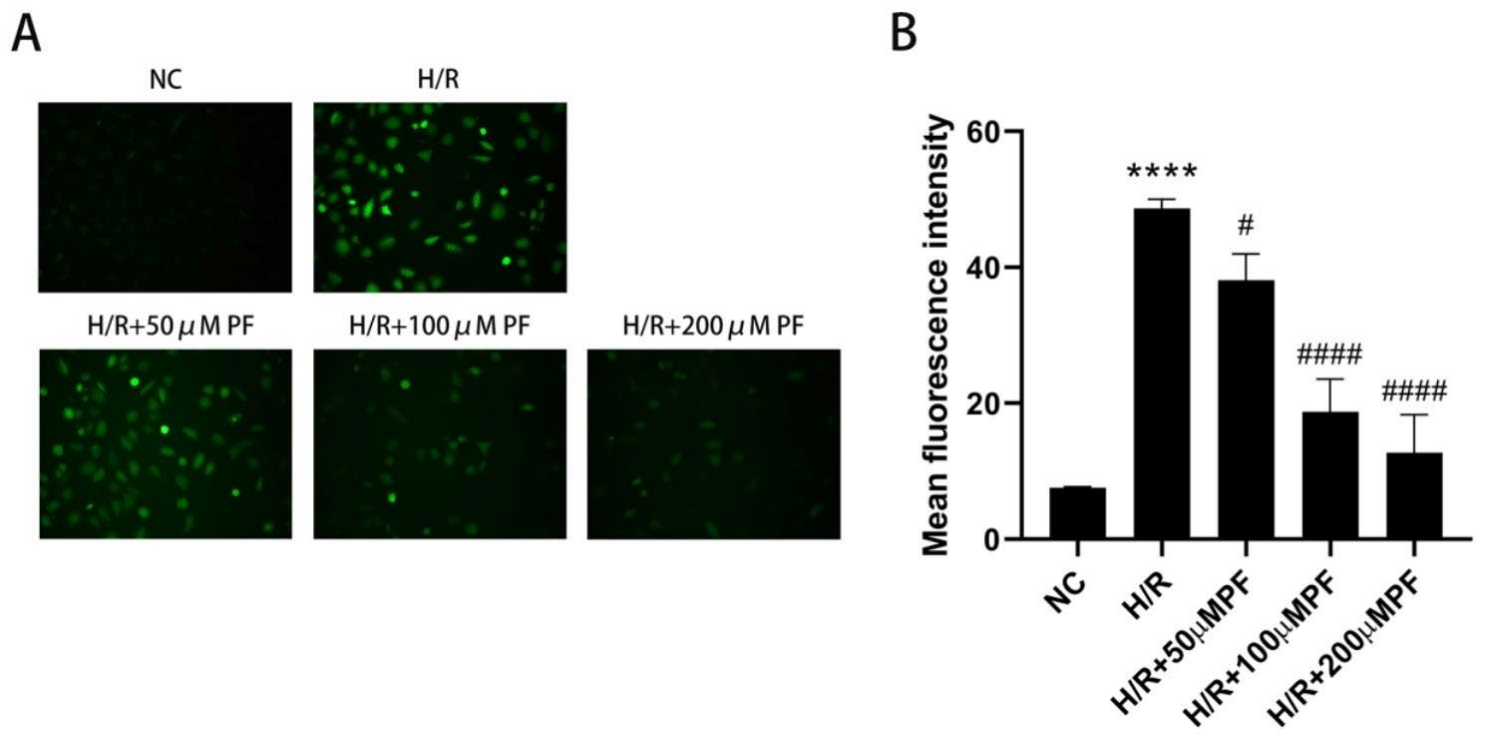
PF attenuated intracellular ROS generation in H/R-induced HK-2 cells. HK-2 cells were pre-treated with 50, 100, and 200µM PF for 4 h, incubated with DMEM/F12 (serum-free culture medium) containing 400µM of COCL_2_ for 24 h to mimic a hypoxia environment, and then subjected to reoxygenation treatment for 4 h. The intracellular ROS levels were directly observed under a fluorescence microscope after DCFH-DA loading. (**A**) Intracellular ROS were detected by fluorescence microscopy. (**B**) The results are presented as the mean fluorescence intensity as analyzed by Image J software. Data are expressed as the mean ± SD (n = 3). ****p < 0.0001, in comparison with the NC group. #*p* < 0.05, ####*p* < 0.0001, in comparison with the H/R group. NC, normal control, H/R, hypoxia/reoxygenation, PF, paeoniflorin, SD, standard deviation

Continuous intracellular ROS generation is an important step that induces oxidative damage during renal H/R injury. To investigate the antioxidative role of PF in HK-2 cells with H/R treatment, the ROS levels were evaluated using a DCF fluorescence probe. HK-2 cells which undergoing H/R treatment revealed higher DCF fluorescence (Fig. 2A), indicating the generation of more ROS. Compared with the H/R group, the ROS levels in three different concentrations (50 µM, 100 µM, and 200 µM) of the PF group were significantly decreased (*p* < 0.05), among which 200-µM PF exhibited the lowest ROS level (Fig. 2B, p < 0.01). The results indicated that PF pretreatment could markedly inhibit ROS in HK-2 cells with H/R injury in a concentration-dependent manner. Thus, 200 µM was selected as the optimal concentration of PF.

### PF inhibited apoptosis and decreased HIF-1α protein expression in HK-2 cells with H/R injury

**Fig. 3:**
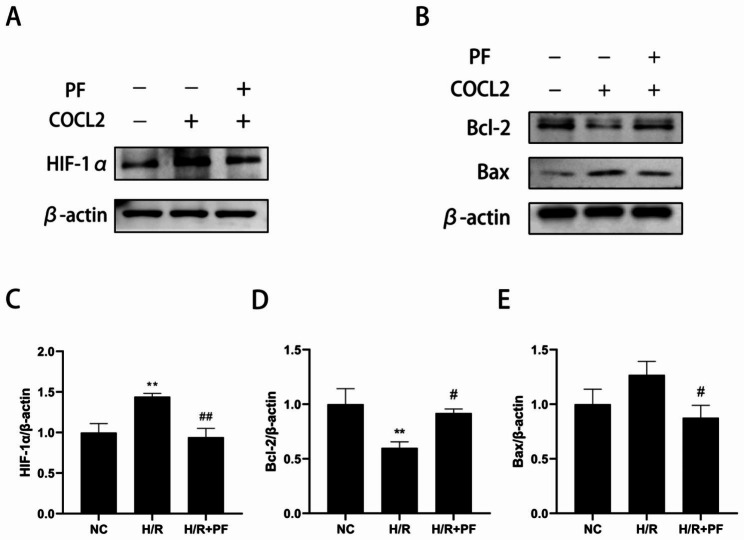
PF decreased apoptosis and the expression of HIF-1α in H/R-induced HK-2 cells. HK-2 cells were pre-processed by 200µM of PF for 4 h and then subjected to H/R treatment as mentioned earlier. The grouping of blots cropped from different parts of the same gel. (**A**) The protein expression of HIF-1α was detected by Western blotting. (**B**) The protein expression of Bax and Bcl-2 were measured by Western blotting. (**C–E**) The changes in the protein expression of HIF-1α, Bax, and Bcl-2 were standardized to those of β-actin. The values are expressed as the mean ± SD (n = 3). **p < 0.01, in comparison with the NC group. ##*p* < 0.01, in comparison with the H/R group. NC, normal control, H/R, hypoxia/reoxygenation, PF, paeoniflorin, SD, standard deviation

Hypoxia-inducible factor-1α (HIF-1α) is a protein and is upregulated under a hypoxic environment. To confirm whether COCL_2_ treatment induced oxidative stress by creating hypoxic conditions, we performed a western blotting assay to evaluate the protein expression of HIF-1α. Compared with the NC group, HIF-1α was significantly upregulated in the H/R group, which was attenuated by PF (Fig. 3A and C; *p* < 0.01). Furthermore, we evaluated the apoptosis relative protein expression and found a marked increase in the expression of anti-apoptotic protein Bcl-2 in the H/R + PF group and a significant reduction in the expression of pro-apoptotic protein Bax (Fig. 3B, D, and E; *p* < 0.05). These results indicated that PF could inhibit H/R injury-induced apoptosis in HK-2 cells.

### PF increased SOD activity and decreased MDA

**Fig. 4:**
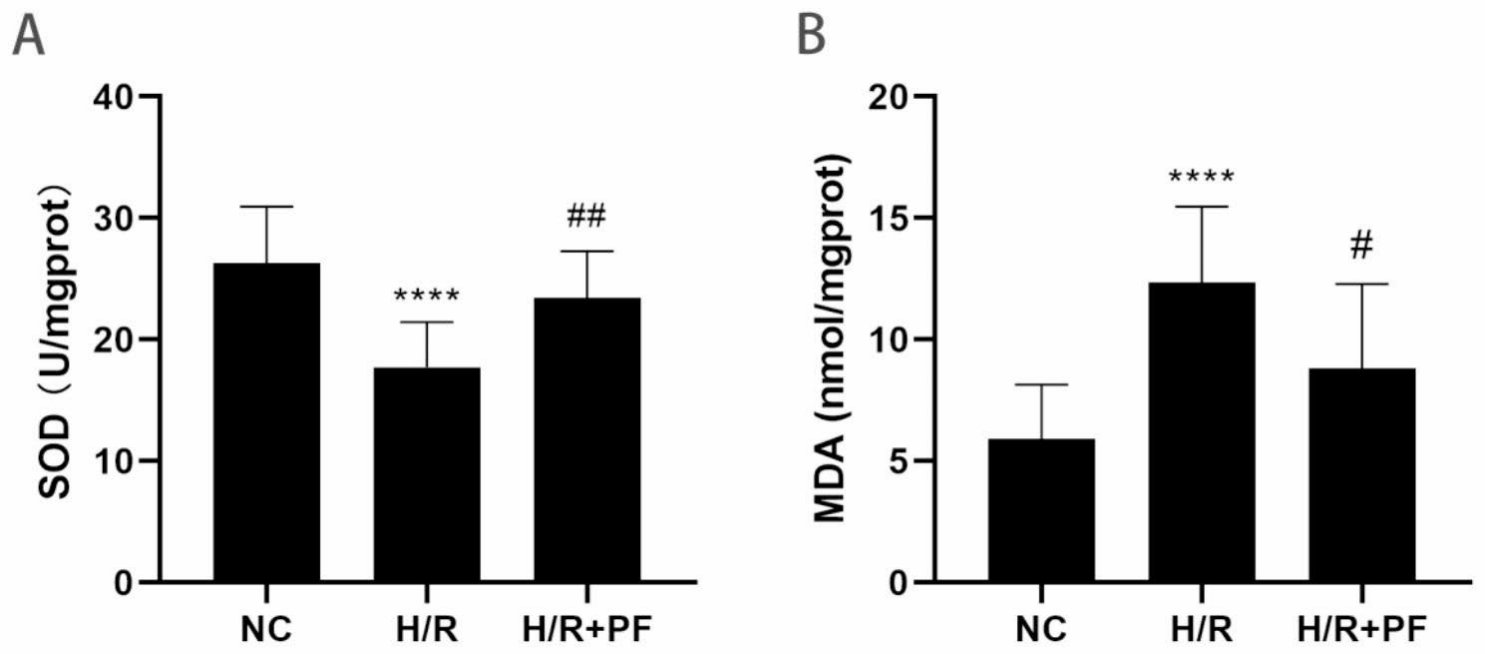
PF increased the SOD activity and reduced the MDA level. HK-2 cells were pre-processed by 200µM of PF for 4 h and then subjected to H/R treatment as mentioned earlier. (**A**) The SOD activity was detected by using commercial assay kits. (**B**) The MDA level was determined by using commercial assay kits. The values are expressed as the mean ± SD (n = 11). ****p < 0.0001, in comparison with the NC group. ##*p* < 0.01, in comparison with the H/R group. NC, normal control, H/R, hypoxia/reoxygenation, PF, paeoniflorin, SD, standard deviation

SOD is a group of antioxidant enzymes that participate in the antioxidative defense against highly reactive superoxide radicals. The MDA levels reflect the level of lipid peroxidation in an organism. We found that, when compared with the NC group, the SOD activity was decreased and the MDA levels were increased in the H/R group (Fig. 4A, B; *p* < 0.05). However, PF could significantly improve the SOD activity (*p* < 0.01) and attenuate the MDA levels (*p* < 0.05). These results indicate that PF exerts a protective function by inhibiting oxidative stress.

### PF downregulated Keap1 expression, but upregulated Nrf2 and HO-1 expression

**Fig. 5:**
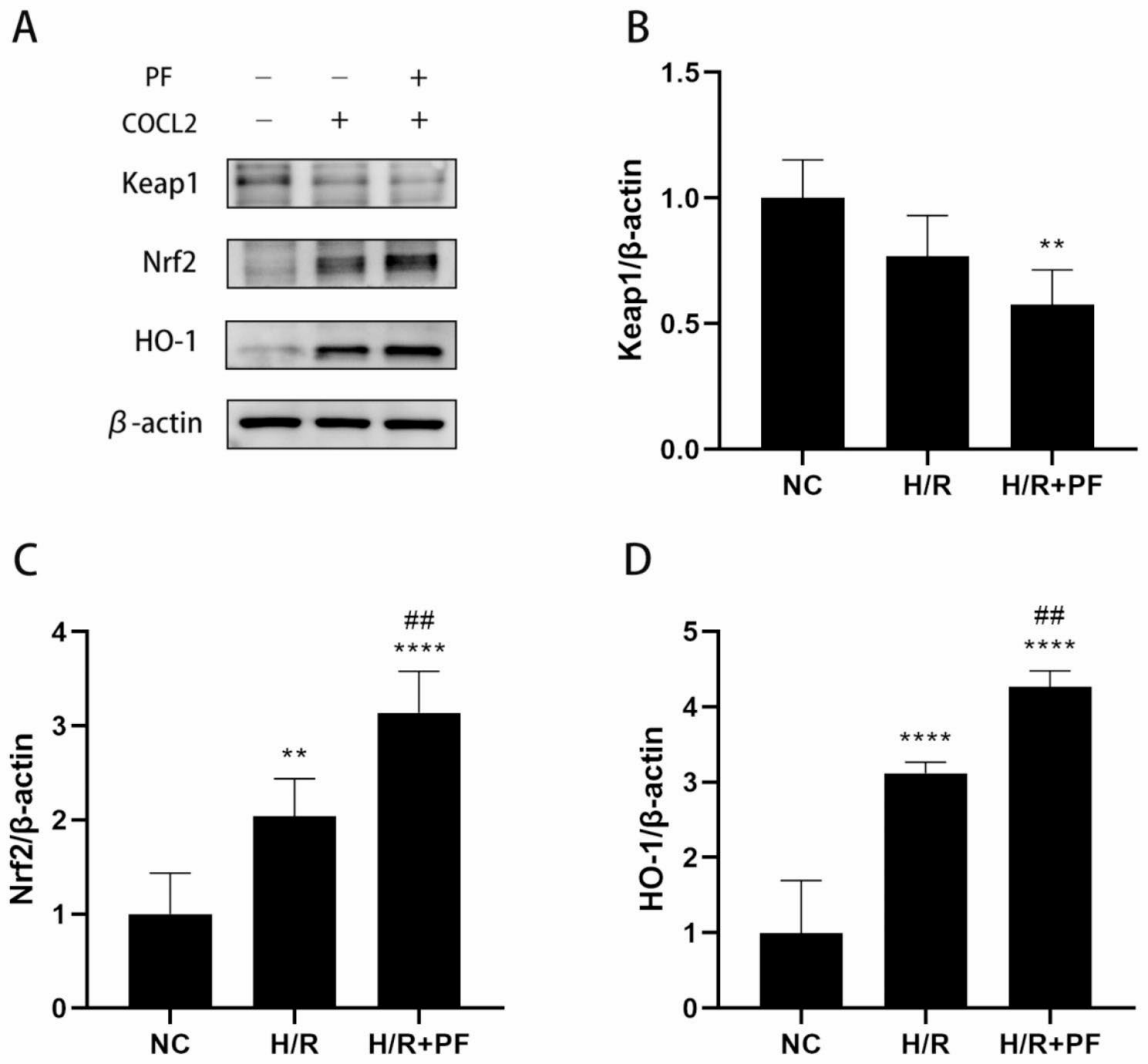
The role of PF in the regulation of the Keap1/Nrf2/HO-1 pathway. HK-2 cells were pre-processed by 200µM of PF for 4 h and then subjected to H/R treatment as mentioned earlier. The grouping of blots cropped from different parts of the same gel. (**A**) The expression of Keap1, HO-1, and Nrf2 were measured by Western blotting. (**B–D**) The changes in the protein expression of Keap1, HO-1, and Nrf2 were standardized to those of β-actin. The data are expressed as the mean ± SD (n = 5). **p < 0.01, ****p < 0.0001, in comparison with the NC group. ##*p* < 0.01, in comparison with the H/R group. HO-1, heme oxygenase 1, Nrf2, nuclear factor erythroid 2-related factor 2, Keap1, Kelch-like ECH-associated protein 1, NC, normal control, H/R, hypoxia/reoxygenation, PF, paeoniflorin, SD, standard deviation

Nrf2 is an important regulator of the antioxidative system, which maintains the balance of the oxidative/antioxidative enzyme expression. Keap1 negatively regulates Nrf2 by binding to Nrf2 and promoting its degradation in normoxia [16]. To elucidate the underlying mechanism of the protective effect of PF on renal H/R injury, we evaluated the relative protein expression of the Keap1/Nrf2/HO-1 signaling pathway. The results showed that the expression of Keap1 was downregulated, whereas the expression of HO-1 and Nrf2 was upregulated (Fig. 5; *p* < 0.01) in HK-2 cells undergoing H/R injury. Interestingly, compared with the H/R group, pretreatment with PF further downregulated the Keap1 expression but upregulated the HO-1 and Nrf2 expression (*p* < 0.01).

### PF increased Nrf2 nuclear translocation and promoted HO-1 expression

**Fig. 6:**
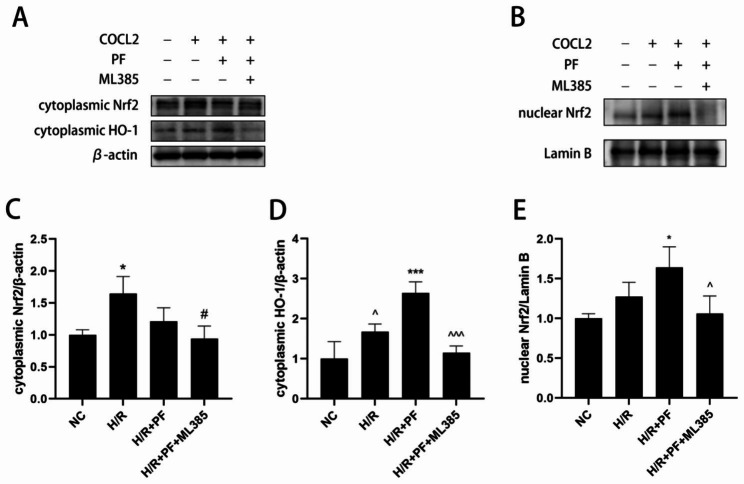
PF advanced the translocation of Nrf2 to the nucleus. HK-2 cells were treated with ML385 (5µM) for 24 h before PF pretreatment and H/R treatment. The grouping of blots cropped from different parts of the same gel. (**A**) The expression of cytoplasmic Nrf2 and HO-1 were measured by Western blotting. (**B**) Western blotting analysis of the expression of nuclear Nrf2. (**C–E**) The changes in the cytoplasmic Nrf2 and HO-1 protein expression were normalized to those of β-actin, and the change in the protein expression of nuclear Nrf2 was normalized to that of lamin B. The values are expressed as the mean ± SD (n = 3). *p < 0.05, ***p < 0.001, in comparison with the NC group. #*p* < 0.05, in comparison with the H/R group. ^*p* < 0.05, ^^^*p* < 0.001, in comparison with the H/R + PF group. NC, normal control, H/R, hypoxia/reoxygenation, PF, paeoniflorin, SD, standard deviation

Under normoxia, Nrf2 binds to Keap1 in the cytoplasm and stays in a low expression state. However, after ROS stimulation, Nrf2 escapes from the control of Keap1, translocates into the nucleus and promotes the expression of an antioxidative enzyme. The expression of cytoplasmic Nrf2 was distinctly upregulated in the H/R group when compared to that in the NC group (Fig. 6A, C; *p* < 0.05). Additionally, the expression of nuclear Nrf2 (Fig. 6B, E) and cytoplasmic HO-1 (Fig. 6A, D) were markedly increased (*p* < 0.05) after PF pretreatment, suggesting that PF could increase Nrf2 nuclear translocation and further promote HO-1 expression. Contrastingly, a selective Nrf2 inhibitor (ML385) could effectively prevent PF from initiating the Nrf2/HO-1 signaling pathway (Fig. 6A–E).

### Nrf2 inhibitor reversed the protection of PF in H/R-induced HK-2 cells

**Fig. 7:**
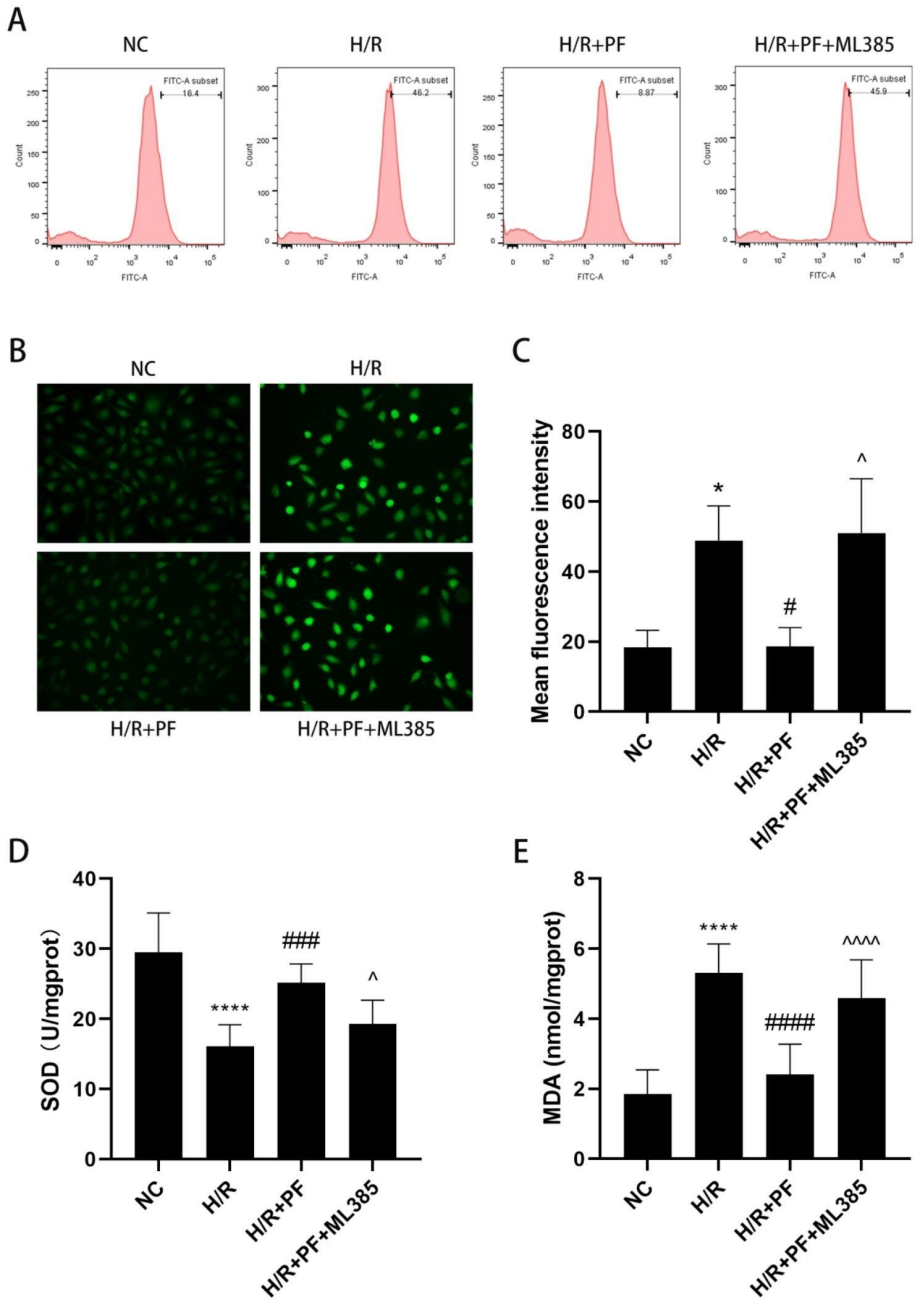
Nrf2 inhibitor abolished the protection of PF on H/R-induced HK-2 cells. (**A**) The intracellular ROS level was measured by flow cytometry. (**B**) The intracellular ROS level was detected by using fluorescence microscopy. (**C**) The data shown are the mean fluorescence intensities analyzed by using the Image J software (n = 3). (**D**) The SOD activity was detected by using a commercial assay kit. € The MDA level was determined by using commercial assay kits. The values were expressed as the mean ± SD (n = 9). *p < 0.05, ****p < 0.0001, in comparison with the NC group. #*p* < 0.05, ###*p* < 0.001, ####*p* < 0.0001, in comparison with the H/R group. ^*p* < 0.05, ^^^^*p* < 0.0001, in comparison with the H/R + PF group. NC, normal control, H/R, hypoxia/reoxygenation, PF, paeoniflorin, SD, standard deviation

To further elucidate the mechanism of PF in protecting H/R-induced HK-2 cells from oxidative damage, we inhibited Nrf2 using ML385, a selective Nrf2 inhibitor, and evaluated intracellular ROS levels, SOD activity, and MDA levels. Pretreatment with ML385 increased the intracellular ROS levels (Fig. 7A–C) and MDA levels (Fig. 7E), but decreased the SOD activity (Fig. 7D) when compared with those in the H/R + PF group. The results suggested that Nrf2 inhibition could successfully reverse the antioxidative function of PF on HK-2 cells with H/R injury (*p* < 0.05). When combined with the results shown in Fig. 6, our hypothesis that PF protects HK-2 cells from H/R injury through the Nrf2/HO-1 signaling pathway can be proven.

## Discussion

Because AKI is a common clinical problem worldwide, an effective treatment is required that can treat or delay the development of this disease. However, no certain medicine can prevent AKI development owing to its complicated pathology. Because PF exerts various biological activities in preventing various diseases, we established a H/R model using human kidney proximal tubule epithelial cells to investigate whether PF exerts positive effects on AKI. In this study, we demonstrated that PF could alleviate the oxidative damage in HK-2 cells with H/R injury, and with increasing the activity of SOD, decreasing the levels of ROS and MDA. Furthermore, PF could increase the viability of HK-2 cells with H/R injury and inhibit apoptosis by upregulating the Bcl-2 expression and decreasing Bax. Furthermore, PF significantly promoted Nrf2 nuclear translocation and increased the HO-1 protein levels. The inhibition of Nrf2 considerably reversed the renal protective ability of PF.

COCL_2_ is a chemical hypoxia inducer that has been used to mimic the hypoxia state. COCL_2_ can increase HIF-1α and also trigger the accumulation of ROS [19]. In normoxia, HIF-1α undergoes hydroxylation and facilitates ubiquitination and proteasomal degradation via a specific HIF-prolyl hydroxylase domain protein (PHD), which is upregulated in a hypoxic environment [20]. HIF-1α-induced HO-1 expression showed that HIF-1α participates in the activation of the antioxidative defense system [21]. In this study, we used COCL_2_ and fresh completed culture medium to construct a H/R injury model using HK-2 cells based on a previous study [22] and demonstrated that the HIF-1α expression was upregulated in the H/R group, which was accompanied by the generation of ROS. However, PF downregulated the expression of HIF-1α and inhibited ROS production in a concentration-dependent manner, which indicating that PF could decrease ROS generation induced by a hypoxia.

Kidney proximal tubule epithelial cells play a key role in renal reabsorption, which requires high ATP levels [4]. However, owing to a COCL_2_-induced hypoxia environment, mitochondrial oxidative phosphorylation does not occur as usual, which further affects ATP production, leading to ROS accumulation. Furthermore, the condition worsens during reoxygenation because of ROS generation [1], [3]. ROS generation is a key indicator of oxidative stress in an organism. SOD and MDA are two classical biomarkers of oxidative stress, representing antioxidative activity and lipid peroxidation, respectively, and their levels are always measured together to investigate the oxidative stress levels [23]. Jing Yu et al. reported that PF protected gamma-radiation-induced human endothelial cells from oxidative damage by reducing the MDA levels, ROS production, and LDH leakage and improved the endogenous antioxidant (SOD) generation [6]. The present results demonstrated reduced SOD activities and high MDA levels in H/R renal cells, unlike those in HK-2 cells, and PF reversed the abovementioned effects as expected. These results indicated that PF prevented HK-2 cells from H/R injuries by reducing the extent of oxidative damage.

Nrf2 is a transcription activator in the nucleus that binds with small Mafs and further combines with AREs to initiate the gene expression of antioxidative enzymes [24]. HO-1 is an antioxidative enzyme responsible for several antioxidant, anti-inflammatory, and antiapoptotic pathways [23]. Accumulating evidence shows that the Keap1/Nrf2/ARE signaling pathway plays a role in maintaining redox homeostasis. Yang et al. found that PF induced the Keap1/Nrf2 pathway and increased the downstream antioxidative enzyme levels in high-glucose-induced RSC96 cells [25]. Nrf2 activation mitigated kidney injuries caused by oxidative stress [26]. Moreover, CDDO–imidazolide treatment improved the renal functions after I/R-induced AKI in mice [18].

The Kelch domain of Keap1 interacts with the Neh2 domain of Nrf2 and binds with the actin cytoskeleton in the cytoplasm. Therefore, most Nrf2 proteins enter ubiquitination degradation and a few translocate to the nucleus, thereby maintaining a low basal level [24]. Nrf2 phosphorylation by protein kinase C and changes in the cysteine residue of Keap1 are probably related to Nrf2 liberation [24]. *Keap1* expression was downregulated and NQO1 and HO-1 mRNA expression was upregulated in H/R-stimulated hepatocytes [27]. Consistent with previous results, the present results revealed that PF downregulated *Keap1* expression and upregulated *HO-1* and *Nrf2* expression in the H/R group, which was more pronounced after PF treatment. These results implied that Nrf2 was induced to dissociate from Keap1 when the organism was under oxidative stress and that PF treatment significantly increased this dissociation. Furthermore, the accumulation of released Nrf2 increased the nuclear translocation of Nrf2. PF treatment increased the nuclear translocation of Nrf2 and upregulated the gene expression of antioxidative enzymes [8], [28]. The present results showed that the nuclear *Nrf2* was significantly upregulated in the H/R + PF group, with increased *HO-1* expression in the cytoplasm, whereas the expression of cytoplasmic *Nrf2* was upregulated in the H/R group only. Oxidative defense is induced in the early stage when an organism is stimulated by external factors. However, this adaptive response is insufficient to resist the continuous damage caused by H/R injuries. PF treatment mitigated this damage by enhancing Nrf2 nuclear translocation. Thus, we believe that PF indirectly or directly activates Nrf2 phosphorylation or disrupts the Keap1 conformational structure to facilitate Nrf2 nuclear translocation. However, this points need validation through further research.

To confirm that PF prevented HK-2 cells from H/R injuries by inhibiting oxidative damage via Keap1/Nrf2/ARE pathway activation, we administered ML385 before PF treatment in HK-2 cells with H/R injuries and found that Nrf2 inhibition markedly reversed the renal protection effect of PF by increasing the MDA levels and alleviating ROS and SOD activities. Furthermore, ML385 inhibited Nrf2 nuclear translocation and reduced *HO-1* expression. These results were consistent with previous one suggesting that transfection with Nrf2 siRNAs hindered protection by PF in gamma-radiation-impaired human EA.hy926 cells and reduced HO-1 mRNA and protein levels [6]. Furthermore, a study revealed that ML385 partially blocked the function of lycopene in Nrf2/HO-1 pathway regulation in H/R-induced hepatic cells [29].

In summary, PF exerted renal protection by inhibiting oxidative damage, and these effects were essentially dependent on the Keap1/Nrf2/HO-1 pathway activation. The limitation of this study is that we did not use an in vivo H/R model to confirm the relationship between PF treatment and the Keap1/Nrf2/HO-1 pathway in AKI. Hence, future studies should conduct animal experiments to explore the effects of PF on AKI.

## Conclusions

In conclusion, the present study revealed that PF treatment prevented HK-2 cells from H/R injuries and that the protective role of PF may be relevant to Nrf2-mediated antioxidant reactions. Furthermore, the Nrf2/HO-1 pathway is a potential target for treating H/R-induced oxidative stress. The antiapoptotic and antioxidant role of PF may have applications in the clinical therapy of I/R-induced AKI.

### Electronic supplementary material

Below is the link to the electronic supplementary material.

Supplementary Material 1

Supplementary Material 2

Supplementary Material 3

Supplementary Material 4

Supplementary Material 5

Supplementary Material 6

Supplementary Material 7

Supplementary Material 8

Supplementary Material 9

Supplementary Material 10

Supplementary Material 11

Supplementary Material 12

Supplementary Material 13

Supplementary Material 14

Supplementary Material 15

## Data Availability

The datasets used and/or analyzed during the current study available from the corresponding author on reasonable request.
