# Supplementary material for: Paeoniflorin alleviates hypoxia/reoxygenation injury in HK-2 cells by inhibiting apoptosis and repressing oxidative damage via Keap1/Nrf2/HO-1 pathway
Source: BMC Nephrol. 2023 Oct 26;24:314. doi: 10.1186/s12882-023-03366-0 (PMC10601317; doi:10.1186/s12882-023-03366-0)

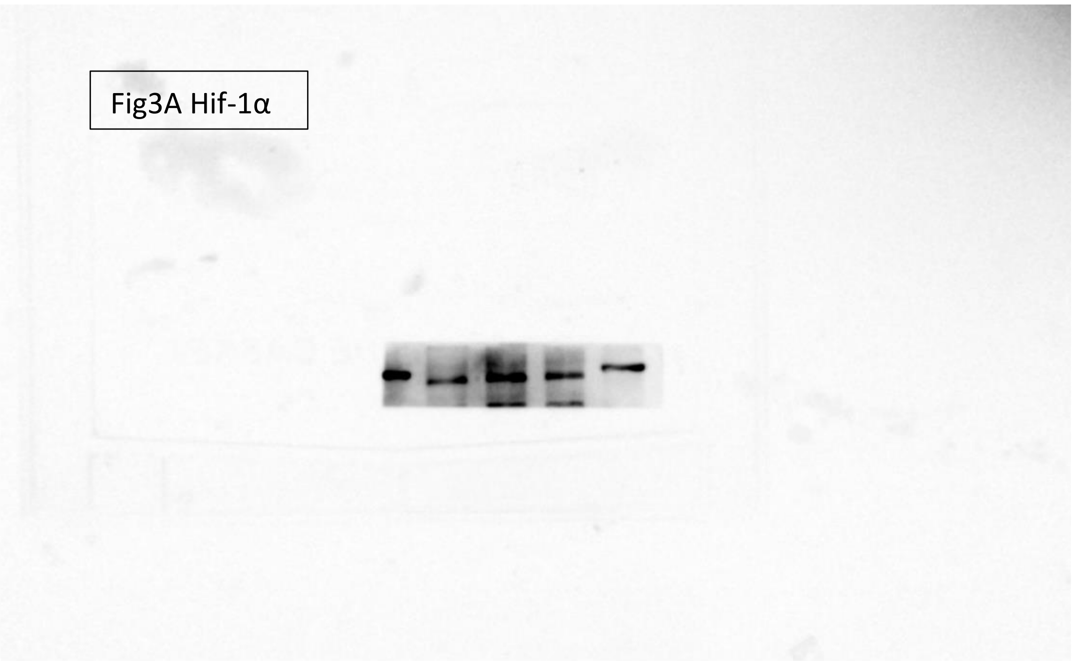
Fig3A Hif-1α

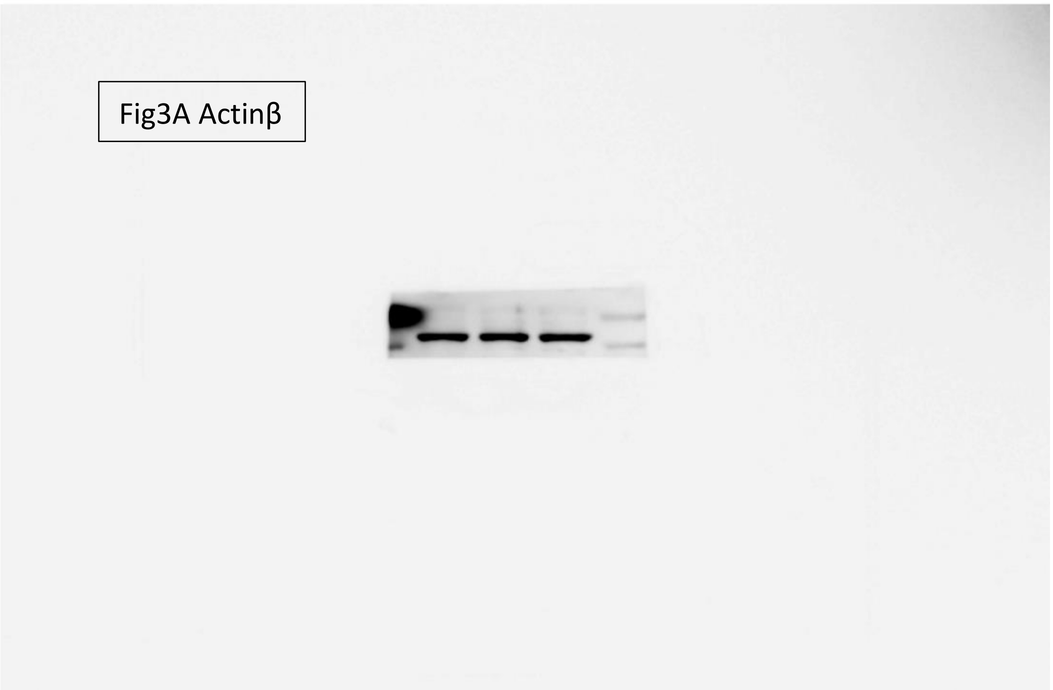
Fig3A Actinβ

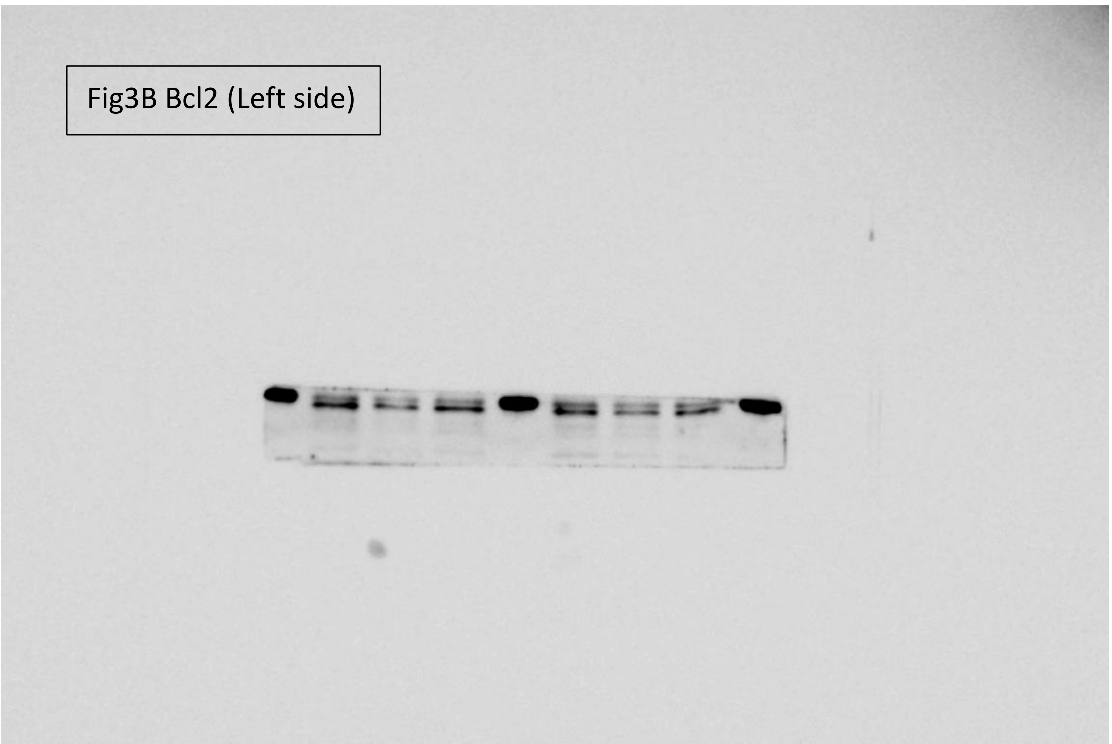
Fig3B Bcl2 (Left side)

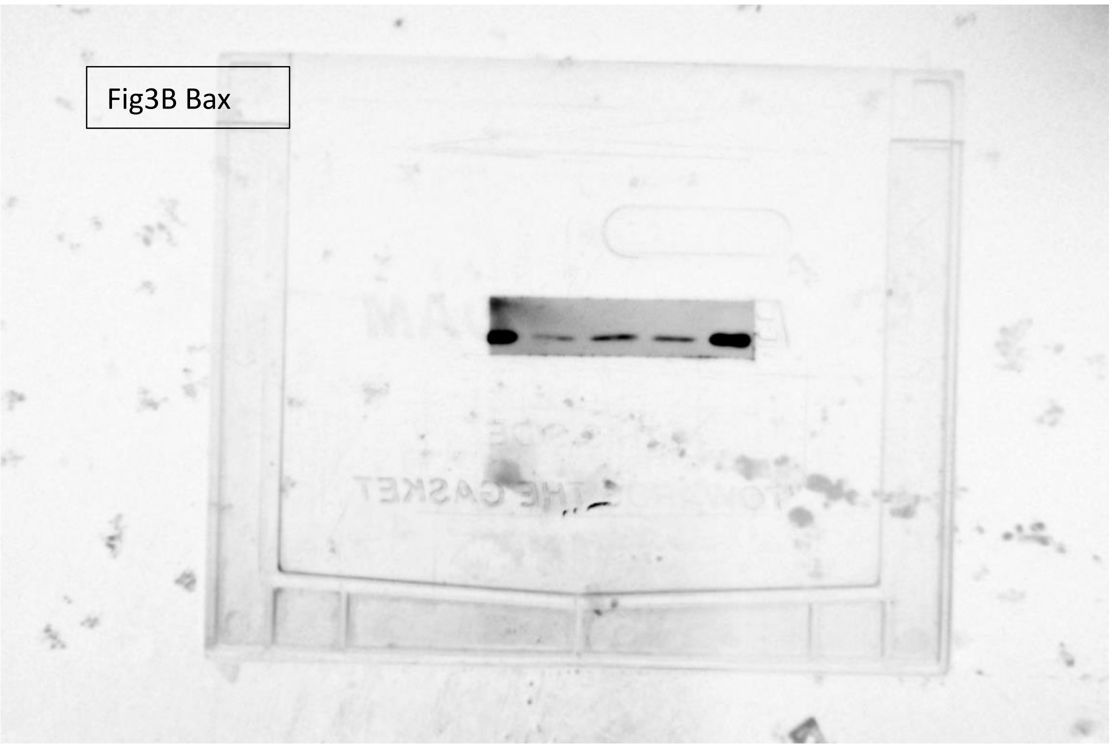
Fig3B Bax

*The edges of the membrane were framed with solid lines.

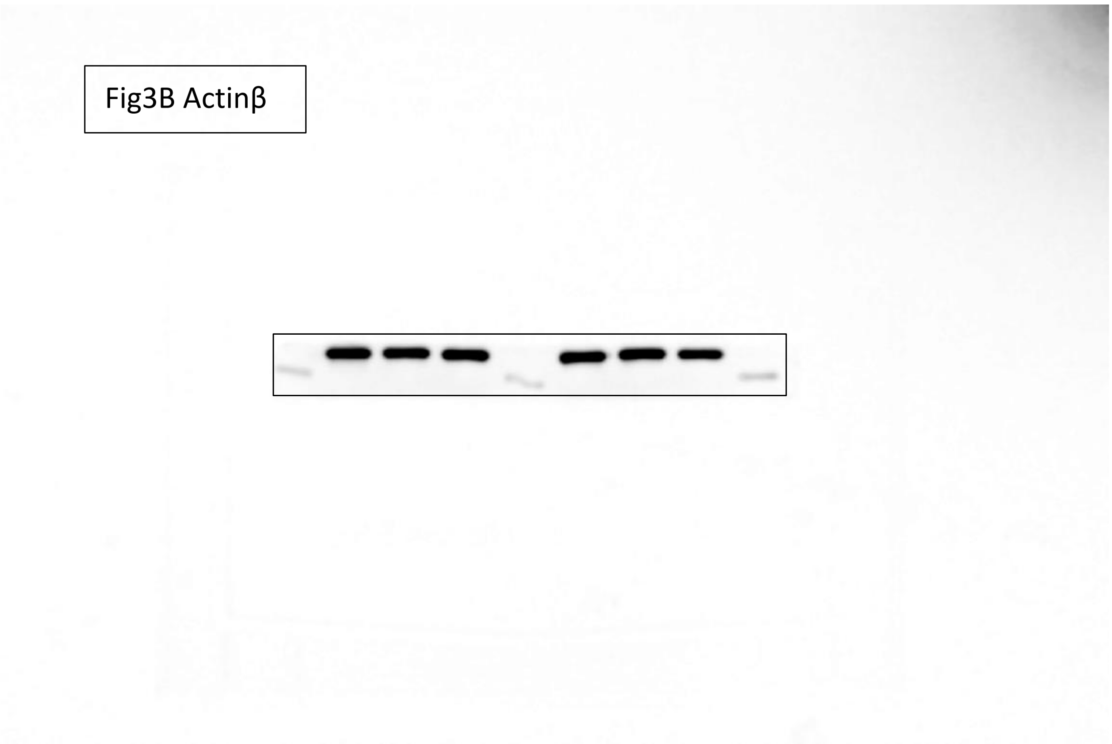

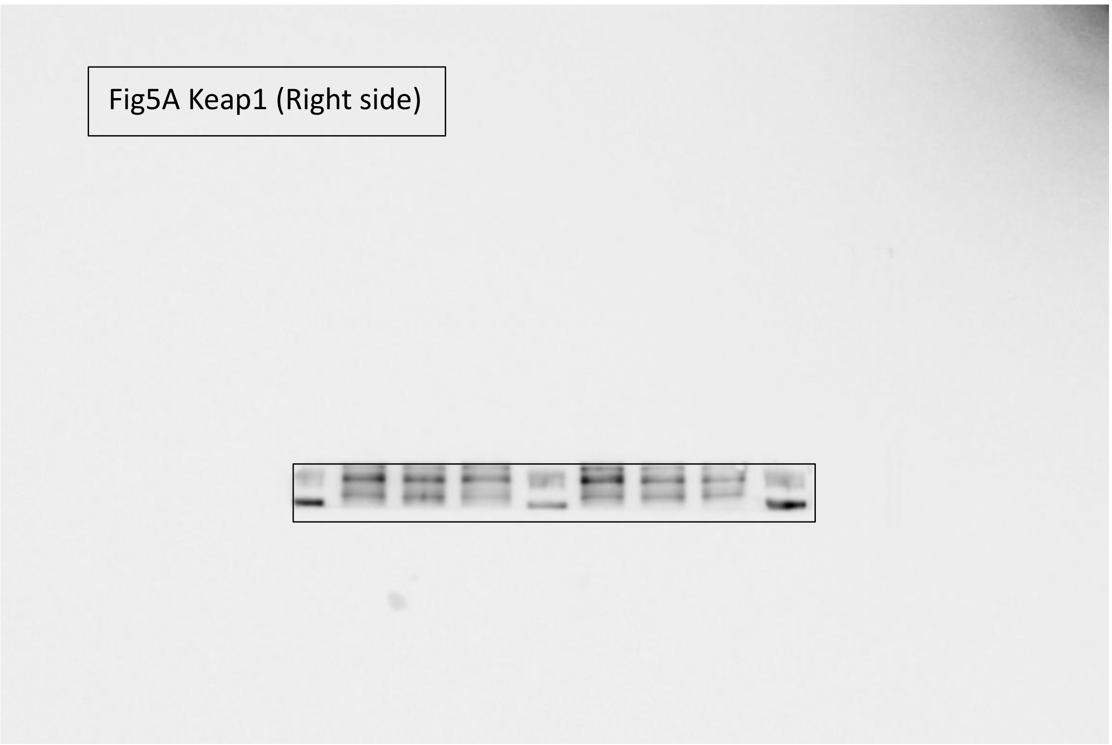

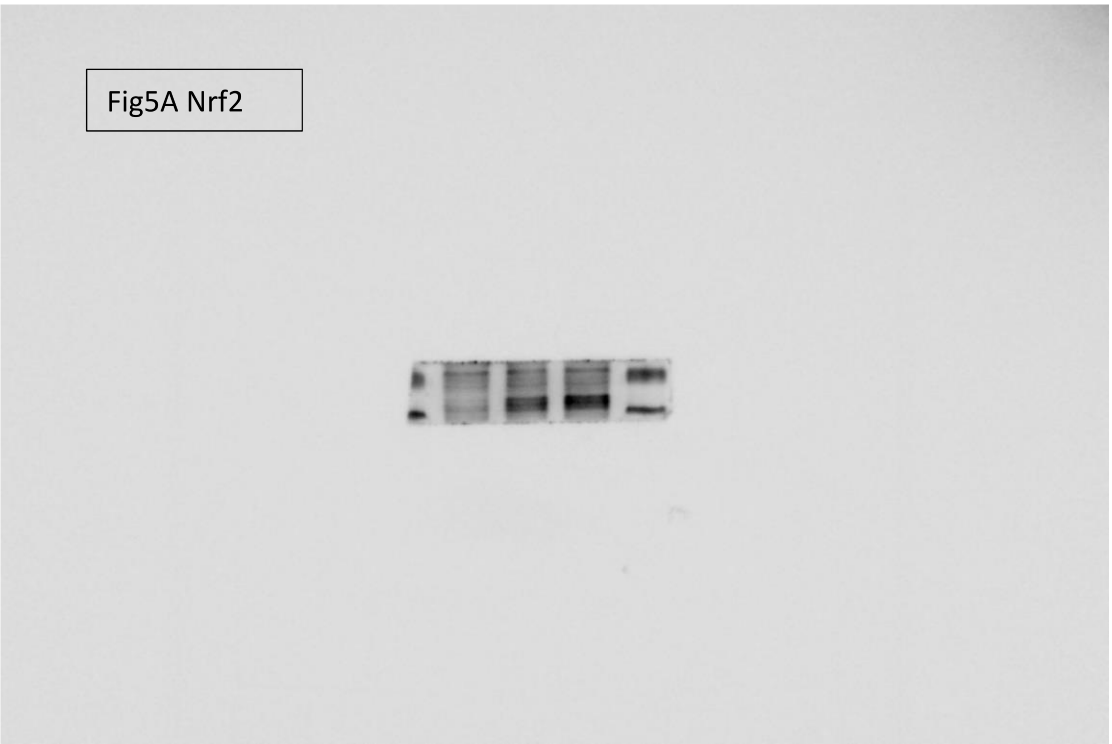
Fig5A Nrf2

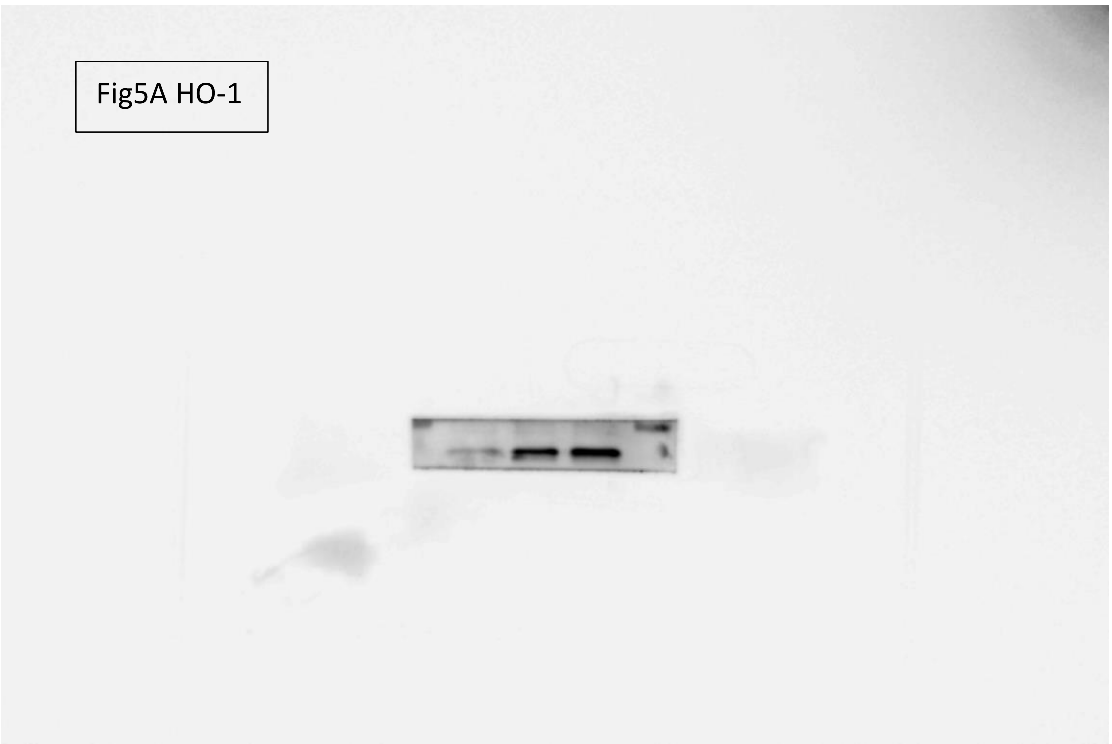
Fig5A HO-1

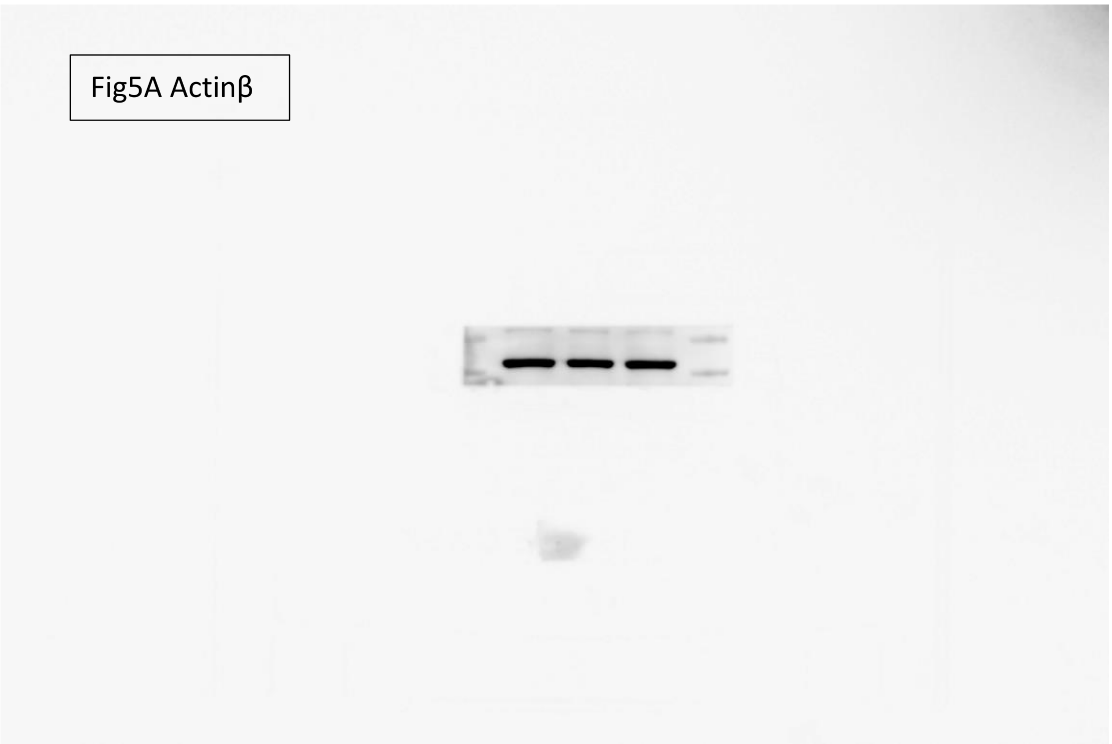
Fig5A Actinβ

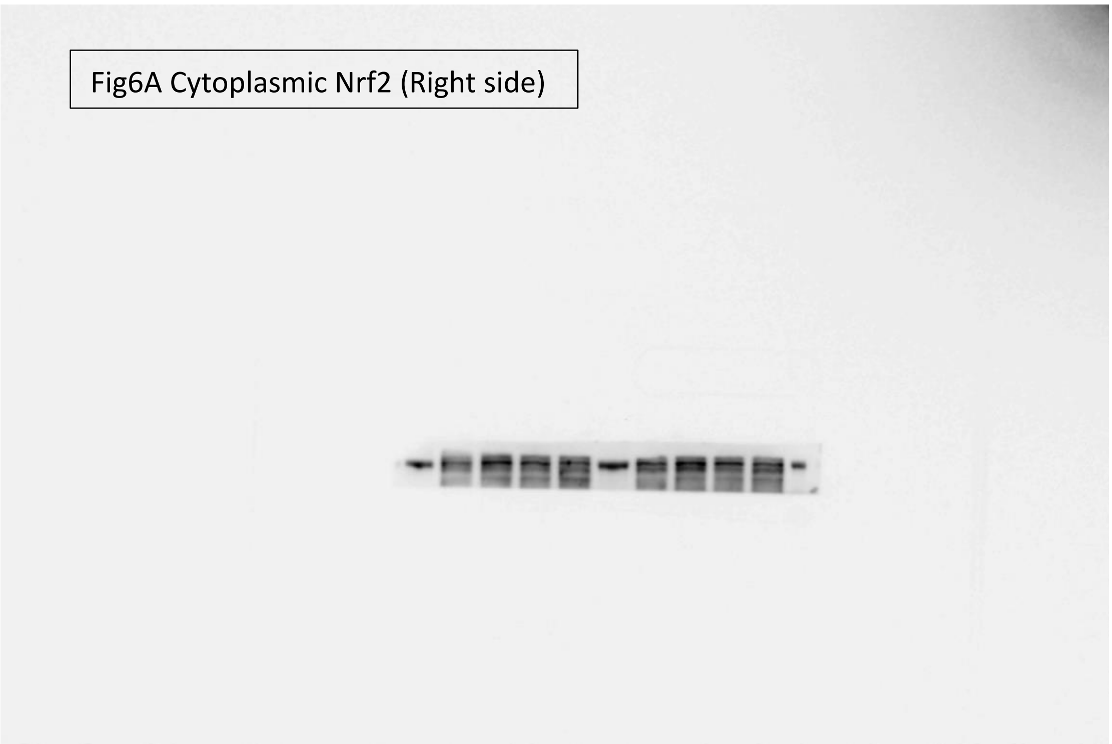
Fig6A Cytoplasmic Nrf2 (Right side)

Fig6A Cytoplasmic HO-1

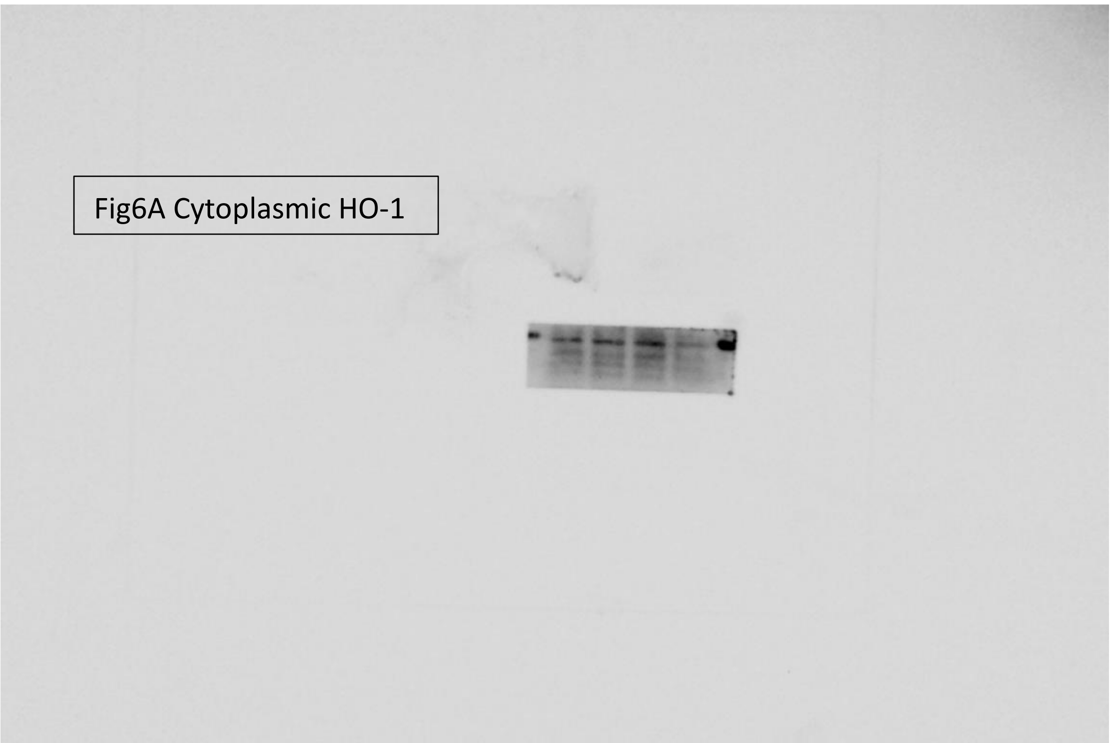

*The edges of the membrane were framed with solid lines.

Fig6A Actinβ (Right side)

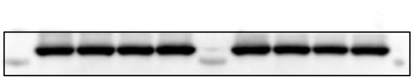

Fig6B Nuclear Nrf2

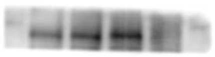

Fig6B LaminB (Right side)

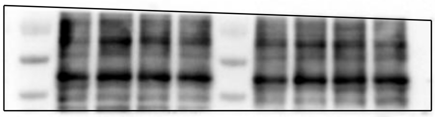

Supplement: Supplementary file 15 — Supplementary Material 15 [file 12882_2023_3366_MOESM15_ESM.pdf]
